# SHIP1 Controls Internal Platelet Contraction and α_IIb_β_3_ Integrin Dynamics in Early Platelet Activation

**DOI:** 10.3390/ijms24020958

**Published:** 2023-01-04

**Authors:** Sonia Severin, Alessandra Consonni, Gaëtan Chicanne, Sophie Allart, Bernard Payrastre, Marie-Pierre Gratacap

**Affiliations:** 1 Institut des Maladies Métabolique et Cardiovasculaire (I2MC), Inserm and Université Toulouse III Paul-Sabatier (UMR-1297), 1 Avenue J. Poulhes, CEDEX 4, 31432 Toulouse, France; 2 Laboratory of Biochemistry, Department of Biology and Biotechnology “L. Spallanzani”, University of Pavia, 27100 Pavia, Italy; 3 Institut Toulousain des Maladies Infectieuses et Inflammatoires (Infinity), Université Toulouse III Paul-Sabatier and Inserm (UMR-1291) and CNRS (UMR-5051), Centre Hospitalier Universitaire Purpan, CEDEX 3, 31024 Toulouse, France; 4 Laboratoire d’Hématologie, Centre de Référence des Pathologies Plaquettaires, Centre Hospitalier Universitaire Rangueil, CEDEX 4, 31432 Toulouse, France

**Keywords:** SHIP1, platelets, integrins, cytoskeleton

## Abstract

The Src homology 2 domain-containing inositol 5-phosphatase 1 (SHIP1) is known to dephosphorylate PtdIns(3,4,5)P_3_ into PtdIns(3,4)P_2_ and to interact with several signaling proteins though its docking functions. It has been shown to negatively regulate platelet adhesion and spreading on a fibrinogen surface and to positively regulate thrombus growth. In the present study, we have investigated its role during the early phase of platelet activation. Using confocal-based morphometric analysis, we found that SHIP1 is involved in the regulation of cytoskeletal organization and internal contractile activity in thrombin-activated platelets. The absence of SHIP1 has no significant impact on thrombin-induced Akt or Erk1/2 activation, but it selectively affects the RhoA/Rho-kinase pathway and myosin IIA relocalization to the cytoskeleton. SHIP1 interacts with the spectrin-based membrane skeleton, and its absence induces a loss of sustained association of integrins to this network together with a decrease in α_IIb_β_3_ integrin clustering following thrombin stimulation. This α_IIb_β_3_ integrin dynamics requires the contractile cytoskeleton under the control of SHIP1. RhoA activation, internal platelet contraction, and membrane skeleton integrin association were insensitive to the inhibition of PtdIns(3,4,5)P_3_ synthesis or SHIP1 phosphatase activity, indicating a role for the docking properties of SHIP1 in these processes. Altogether, our data reveal a lipid-independent function for SHIP1 in the regulation of the contractile cytoskeleton and integrin dynamics in platelets.

## 1. Introduction

The platelet cytoskeleton consists of microtubules, cytoplasmic actin filaments mediating contractile events, and the spectrin-based membrane skeleton that laminates the cytoplasmic side of the plasma membrane regulating both membrane dynamics and lateral distribution of trans-membrane glycoproteins including integrins [1,2,3,4]. Following activation, platelets dramatically reorganize their cytoskeleton to regulate shape change, internal contraction, adhesion, and spreading as well as thrombus growth and fibrin clot retraction [5,6,7,8]. Microtubule dynamics and F-actin reorganization are critical for these processes, which also require the generation of contractile forces through the association of cytoplasmic actin filaments with myosin IIA, the major isoform of myosin II in platelets. Deletion of the *myosin IIA* gene in mouse megakaryocytes leads to macrothrombocytopenia and a defective initial platelet contraction with an absence of clot retraction and an increase in the bleeding time [9].

Cytoskeletal reorganization is highly regulated during platelet activation through numerous signaling processes, many of which are modulated by phosphoinositides. Phosphoinositide 3-kinases (PI3K) and their products, the D3-phosphoinositides, are now established as key players in signal transduction in different cell types including platelets [10,11]. Phosphatidylinositol 3,4,5-*tris*phosphate (PtdIns(3,4,5)P_3_) is a second messenger that facilitates the recruitment of pleckstrin homology domain-containing signaling proteins to the plasma membrane [12]. The Src homology 2 (SH2) domain-containing inositol 5-phosphatase 1 (SHIP1) is a regulator of the PI3K-dependent processes through the transformation of PtdIns(3,4,5)P_3_ into PtdIns(3,4)P_2_ [13,14]. In platelets, SHIP1 is a major phosphatase regulating the levels of PtdIns(3,4,5)P_3_ and PtdIns(3,4)P_2_ following platelet activation [15]. SHIP1 becomes tyrosine phosphorylated and recruited to the cytoskeleton following integrin engagement and the loss of its expression results in an accumulation of PtdIns(3,4,5)P_3_ and a strong decrease in PtdIns(3,4)P_2_ production in stimulated and aggregated platelets [15]. In addition to its inositol polyphosphate 5-phosphatase activity, SHIP1 can participate in signal transduction through its docking protein properties as it contains multiple protein interaction domains [16,17]. SHIP1 has an N-terminal SH2 domain, two NPXY sequences which can bind phosphotyrosine-binding motifs, and a C-terminal proline-rich sequence that binds to several Src homology 3 (SH3) domain-containing proteins [14,18]. SHIP1 has been shown to exert negative effects on signal transduction and cellular functions in the immune system [19,20]; however, it can also play positive roles according to the cell type and biological context [14]. Many of the positive effects of SHIP1 in the different models may be explained through its role in cytoskeletal organization. For instance, in neutrophils, the spatio-temporal localization of SHIP1 has important functional consequences in polarization and motility [21,22].

In platelets, SHIP1 has been shown to negatively regulate adhesion and spreading on fibrinogen-coated surfaces [23]. SHIP1 downregulates sustained activation of integrin α_IIb_β_3_ via its phosphatase activity by blocking the PtdIns(3,4,5)P_3_-dependent inhibition of RAS p21 protein activator 3 (RASA3), the main downregulator of RAS-related protein (RAP) 1B, catalyzing the hydrolysis of RAP1B-GTP to RAP1B-GDP [24,25,26]. Thus, in SHIP1-deficient platelets [27] or GAP-deficient RASA3 platelets [24], α_IIb_β_3_ integrin is activated in an uncontrolled manner. This results in platelet overspreading on a fibrinogen matrix [23,24] due to the inability of RASA3 to prevent sustained RAP1B signaling and α_IIb_β_3_ activation [25]. On the other hand, SHIP1 positively controls platelet–platelet interactions and thrombus growth as SHIP1-null platelets showed a dramatic decrease in platelet close contacts and thrombus growth in vivo [27]. To further understand SHIP1 function, we have investigated its role following platelet stimulation by the physiological agonist thrombin in suspension and in absence of aggregation. We showed for the first time that SHIP1 is implicated independently of its phosphatase activity in internal platelet contraction and integrin dynamics. SHIP1 is required for RhoA activation and localization of myosin IIA to the cytoskeleton, both required for the formation of efficient actomyosin complexes. SHIP1 interacts with the spectrin-based membrane skeleton, and its deficiency induces a loss of sustained association of integrins to this network together with a strong decrease in integrin clustering at the membrane surface following thrombin stimulation. Finally, we found that RhoA activation, internal platelet contraction, and the association of integrins to the membrane skeleton were insensitive to the inhibition of PtdIns(3,4,5)P_3_ synthesis or SHIP1 phosphatase activity, indicating a role for the docking functions of SHIP1 in these processes. Altogether, our results demonstrate that during platelet activation, sustained interaction of integrins, including α_IIb_β_3_, to the membrane skeleton and their clustering are controlled by the contractile cytoskeleton which is most likely regulated by the docking properties of SHIP1.

## 2. Results

### 2.1. Abnormalities in Cytoskeletal Reorganization and Internal Contraction in SHIP1-Deficient Platelets

**Figure 1:**
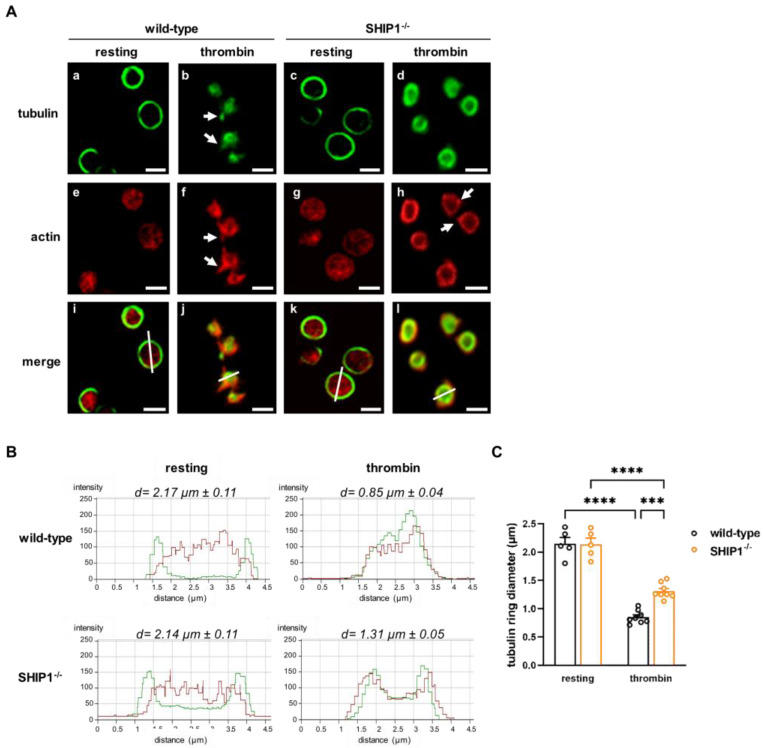
Cytoskeletal reorganization and internal contraction abnormalities in SHIP1-deficient platelets. (**A**) Wild-type or SHIP1-deficient platelets were stimulated or not with thrombin (0.5 IU/mL, 2 min) in suspension under non-aggregating conditions, fixed, and prepared for α-tubulin and actin immunofluorescent staining. Representative confocal images of platelet microtubule (green) and actin cytoskeleton (red) are shown (scale bar = 2 µm). The white arrows point to filopodia. (**B**) Representative panels of tubulin ring (green) diameter and actin cytoskeleton (red) morphometry after line-scan analysis. “d” indicates the mean diameter of the microtubule rings. (**C**) Data of tubulin ring diameter are means ± SEM (*n* = 5–8 independent experiments). *** *p* < 0.001, **** *p* < 0.0001 according to two-way ANOVA test.

To characterize the role of SHIP1 in the control of cytoskeleton dynamics independently of aggregation, we used a confocal microscopy-based morphometric approach [28]. This technique allowed us to study actin and microtubule dynamics in parallel and to evaluate the degree of internal platelet contraction by measuring microtubule ring diameter. We compared wild-type versus SHIP1-null mouse platelets stimulated by thrombin under non-aggregating suspension conditions (presence of the α_IIb_β_3_ blocker integrilin^®^ [29]) (Figure 1). Both in wild-type and SHIP1-null resting platelets, the microtubule coil was at the periphery of the platelets, whereas actin displayed a diffuse labeling pattern. Upon thrombin stimulation, the actin cytoskeleton of wild-type platelets reorganized to display a ring of actin filament bundles with peripheral filopodial extensions (white arrows) and the microtubule ring constricted to the cell center. As previously observed [30,31], some microtubules were seen within filopodial extensions together with actin (white arrows). This reorganization was different in SHIP1-deficient platelets stimulated by thrombin. In these platelets, the microtubule ring was much less condensed, the ring of actin bundles was less constricted, and the actin filopodial extensions were less prominent (Figure 1A). Measurement of the tubulin ring diameter indicated an average of 1.31 ± 0.05 µm for SHIP1-deficient platelets versus 0.85 ± 0.04 µm for wild-type platelets (Figure 1B,C). These data show that SHIP1 deletion strongly impacts the cytoskeletal organization and internal contractile force generation in platelets following thrombin stimulation independently of aggregation.

### 2.2. Lack of SHIP1 Affects RhoA Activation and Destabilizes the Association of Myosin IIA with the Membrane/Cytoskeleton Fraction

**Figure 2:**
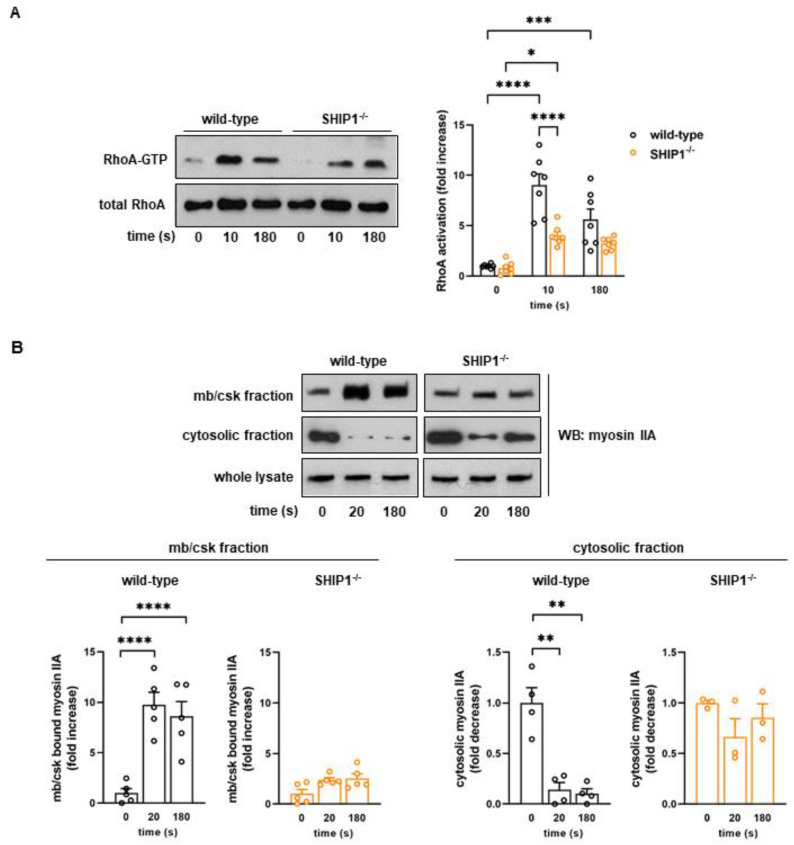
SHIP1 deficiency affects RhoA activation and membrane/cytoskeleton recruitment of myosin IIA following thrombin stimulation. Wild-type and SHIP1-deficient platelets were stimulated with thrombin (0.5 IU/mL) at 37 °C in suspension under non-aggregating conditions. (**A**) Activated form of RhoA was analyzed. Representative Western blots of RhoA from RhoA-GTP pull-down and from whole lysates (total RhoA) are shown. Data are expressed as fold increase in resting (0 s) wild-type and are means ± SEM of 7 independent experiments. * *p* < 0.05, *** *p* < 0.001, **** *p* < 0.0001 according to two-way ANOVA test. (**B**) Platelets were permeabilized by saponin for cytosol depletion. The amount of myosin IIA in the membrane/cytoskeleton (mb/csk) fraction and in the cytosolic fraction was analyzed by Western blotting. Representative Western blots of myosin IIA are shown. Data are expressed as fold increase/decrease in resting (0 s) and are means ± SEM of 3–5 independent experiments. ** *p* < 0.01, **** *p* < 0.0001 according to one-way ANOVA test.

Consistent with the defective internal platelet contraction, RhoA-GTPase, one of the major regulators of the actomyosin contractile apparatus, was much less activated in SHIP1-deficient platelets compared to wild-type platelets following thrombin stimulation (55% ± 4% decrease at 10 s of stimulation) (Figure 2A). To further determine the effect of SHIP1 deficiency on actomyosin formation, we then performed platelet fractionation to isolate the membrane/cytoskeleton (mb/csk) from the cytosol. As shown in Figure 2B, following thrombin stimulation, in the absence of aggregation, myosin IIA was rapidly and massively relocated to the membrane/cytoskeleton of wild-type platelets, while less myosin IIA was detected in the cytosolic fraction (Figure 2B). This process was significantly affected in thrombin-stimulated SHIP1-deficient platelets, with a significant decrease in myosin IIA recruitment to the membrane/cytoskeleton (mb/csk) concomitant to an increase in myosin IIA amount in the cytosolic fraction. These results highlight a critical role for SHIP1 in RhoA activation and in facilitating the association of myosin IIA to the membrane/cytoskeleton (mb/csk) fraction in activated platelets (Figure 2B). Importantly, the total amount of F-actin formed upon thrombin stimulation was not significantly affected by SHIP1 deficiency (Figure S1).

**Figure 3:**
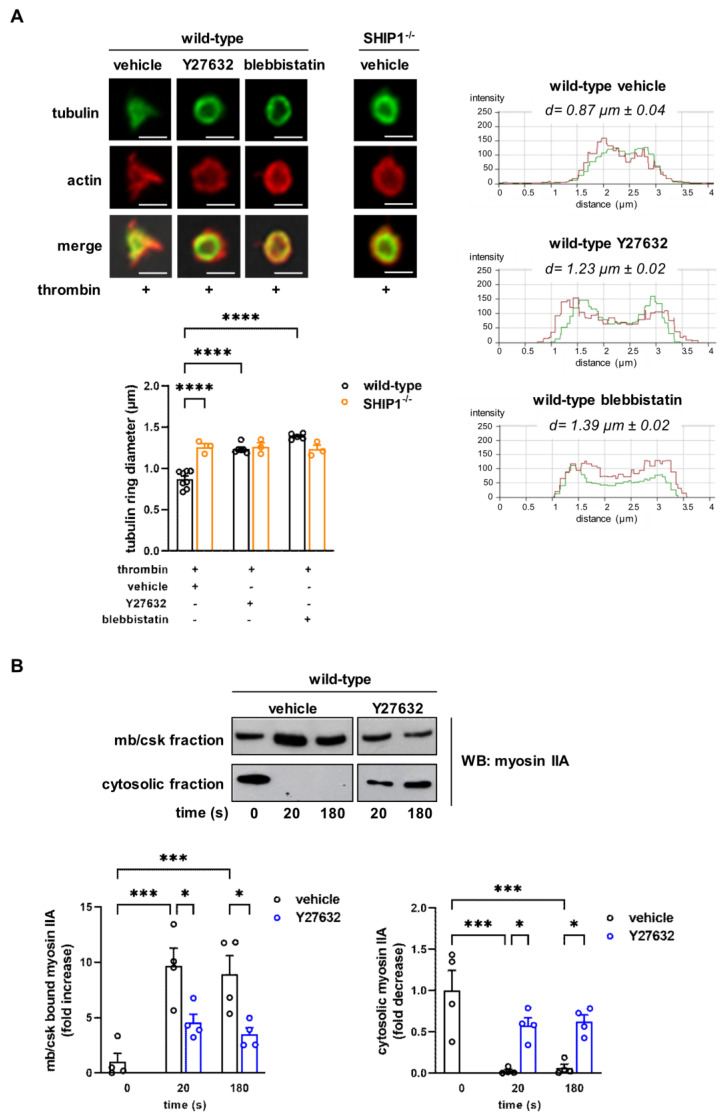
Impact of myosin IIA and Rho-kinase activities on platelet cytoskeletal organization. (**A**) Inhibition of Rho-kinase or myosin IIA did not affect thrombin-induced internal contraction in SHIP1-deficient platelets. Wild-type and SHIP1-deficient platelets were preincubated with vehicle (DMSO), 20 µM Y27632 or 10 µM blebbistatin for 20 min before thrombin stimulation (0.5 IU/mL) for 2 min under non-aggregating suspension conditions. Representative confocal images of platelet microtubule (green) and actin cytoskeleton (red) are shown (scale bar = 2 µm). Representative panels of tubulin ring (green) diameter and the morphometry of actin (red) after line-scan analysis. “d” indicates the mean diameter of the microtubule ring. Data of tubulin ring diameter are means ± SEM (*n* = 3–5 independent experiments). **** *p* < 0.0001 according to two-way ANOVA test. (**B**) The localization of myosin IIA to the membrane cytoskeleton is dependent on Rho-kinase activity. Wild-type platelets were preincubated with vehicle (DMSO) or 20 µM Y27632 for 20 min before thrombin stimulation (0.5 IU/mL) for 2 min under non-aggregating suspension conditions and immediately permeabilized by saponin for cytosol depletion. The amount of myosin IIA in the membrane/cytoskeleton (mb/csk) fraction and in the cytosolic fraction was analyzed by Western blotting. Representative Western blots of myosin IIA are shown. Data are expressed as fold increase/decrease in vehicle-treated resting platelets (0 s) and are means ± SEM of 4 independent experiments. * *p* < 0.05, *** *p* < 0.001 according to two-way ANOVA test.

To analyze if SHIP1-mediated myosin IIA mislocation was related to the dysfunction of the RhoA/Rho-kinase pathway, we investigated the potential implication of Rho-kinase in platelet cytoskeleton organization and internal contraction in response to thrombin using the pharmacological Rho-kinase inhibitor Y27632. Under these conditions, inhibition of Rho-kinase in SHIP1-deficient platelets did not result in an additional decrease in thrombin-induced contraction relative to that observed in vehicle-treated SHIP1-deficient platelets. In contrast, internal platelet contraction was strongly inhibited in wild-type platelets (d = 1.23 ± 0.02 µm) to reach the same level than SHIP1-deficient platelets (d = 1.31 ± 0.05 µm) (Figure 3A and Figure S2). Similar effects were seen in platelets treated with blebbistatin, a myosin IIA inhibitor that blocks its ATPase activity (d = 1.39 ± 0.02 µm, Figure 3A and Figure S2). Consistent with these results, inhibition of the Rho-kinase pathway significantly affected thrombin-induced relocation of myosin IIA to the cytoskeleton in wild-type platelets (Figure 3B), mimicking the effect of an absence of SHIP1 (Figure 2B). Together, these data suggest that SHIP1-mediated regulation of myosin IIA involves the RhoA/Rho-kinase pathway.

### 2.3. SHIP1 Regulates Integrin Anchoring to the Membrane Skeleton and α_IIb_β_3_ Clustering

**Figure 4:**
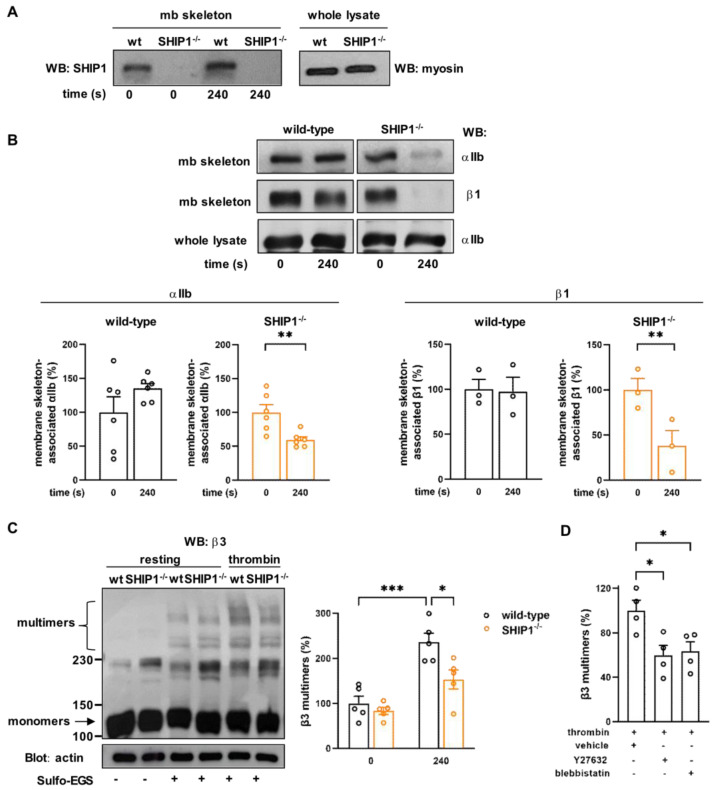
SHIP1 is present at the membrane skeleton and is critical for integrin anchoring to the membrane skeleton and clustering during platelet activation. Platelets from wild-type and SHIP1-deficient mice were stimulated or not with thrombin (0.5 IU/mL) for 4 min under non-aggregating suspension conditions. (**A**,**B**) After isolation of platelet membrane skeleton, the amounts of SHIP1 (**A**), α_IIb_ and β_1_ integrin subunits (**B**) were analyzed by Western blotting. Representative Western blots of SHIP1, α_IIb_, and β_1_ integrin subunits in membrane (mb) skeleton fractions and whole lysates are shown. Data are expressed as a percentage of resting (0 s) and are means ± SEM of 3–6 independent experiments. ** *p* < 0.01 according to unpaired student *t* test. (**C**) After the cross-linking reaction using 0.25 mM Sulfo-EGS cross-linking reagent, with a linker length of 1.6 nm (16 Å), platelet lysates were immunoblotted for β3 integrin subunit. A representative Western blot of β3 integrin subunit is shown. Actin was used as a loading control. Data are expressed as a percentage of resting (0 s) wild-type and are means ± SEM of 5 independent experiments. * *p* < 0.05, *** *p* < 0.001 according to two-way ANOVA test. (**D**) Wild-type platelets were preincubated with vehicle (DMSO), 20 µM Y27632 or 10 µM blebbistatin for 20 min before thrombin stimulation (0.5 IU/mL) for 2 min under non-aggregating suspension conditions and β_3_ multimers were analyzed as in (**C**). Data are expressed as a percentage of vehicle-treated platelets and are means ± SEM of 4 independent experiments. * *p* < 0.05 according to one-way ANOVA test.

Since a significant fraction of SHIP1 belongs to the spectrin-based membrane (mb) skeleton (high-speed Triton X-100-insoluble cytoskeleton) of resting and thrombin-stimulated platelets (32.8% ± 5.7 and 37% ± 6.8, respectively) (Figure 4A), we investigated whether SHIP1 could regulate the association of integrins to this network, thus contributing to efficient integrin function. α_IIb_ and β_1_ integrin subunits, whose surface expression was not modified by SHIP1 deficiency (Figure S3), were associated with the spectrin-based membrane (mb) skeleton in resting platelets from both genotypes. However, the stability of these associations was impaired in SHIP1-null platelets specifically following thrombin stimulation (49.2% ± 5.7% reduction for α_IIb_ and 77% ± 11.4% reduction for β_1_) (Figure 4B). These results show that SHIP1 is required for the stable association of integrins containing α_IIb_, including α_IIb_β_3_, and β_1_ subunits to the membrane skeleton during platelet activation.

We then investigated whether the destabilization of integrins from the spectrin-based membrane skeleton of activated SHIP1-deficient platelets is correlated with a defective integrin clustering. To answer this question, we monitored α_IIb_β_3_ oligomerization (clustering) by using Sulfo-EGS, a reagent cross-linking proteins that fall within a distance of 1.6 nm (16 Å) of each other [32]. In resting platelets, some oligomerization of α_IIb_β_3_ was observed in wild-type and SHIP1-deficient platelets, as shown by the presence of multimers by Western blotting (Figure 4C). However, following activation, the increase in multimer formation observed in wild-type platelets was significantly affected in SHIP1-null platelets (Figure 4C), showing that fewer α_IIb_β_3_ integrins were able to organize in clusters in the absence of SHIP1. It is noteworthy that our conditions of platelet activation (presence of integrilin to avoid aggregation [29]) precluded ligand binding to α_IIb_β_3_, indicating that this step was dispensable for α_IIb_β_3_ clustering, as previously shown in other studies [33].

We then checked whether the actomyosin contractile cytoskeleton, regulated by SHIP1, would contribute to α_IIb_β_3_ integrin clustering. To evaluate this possibility, wild-type platelets were treated with the myosin IIA inhibitor (blebbistatin) or the Rho-kinase inhibitor (Y27632). As shown in Figure 4D, both inhibitors mimicked the phenotype observed in SHIP1-null platelets on integrin α_IIb_β_3_ cluster formation. Altogether, these results demonstrate that SHIP1 is a component of the platelet membrane skeleton and identify SHIP1 as a regulator of α_IIb_β_3_ integrin dynamics and cluster formation following thrombin stimulation, likely via the regulation of the actomyosin contractile cytoskeleton.

### 2.4. Accumulation of PtdIns(3,4,5)P_3_ in SHIP1-Deficient Platelet Is Dispensable for Akt Phosphorylation, RhoA Activation, and Internal Platelet Contraction

**Figure 5:**
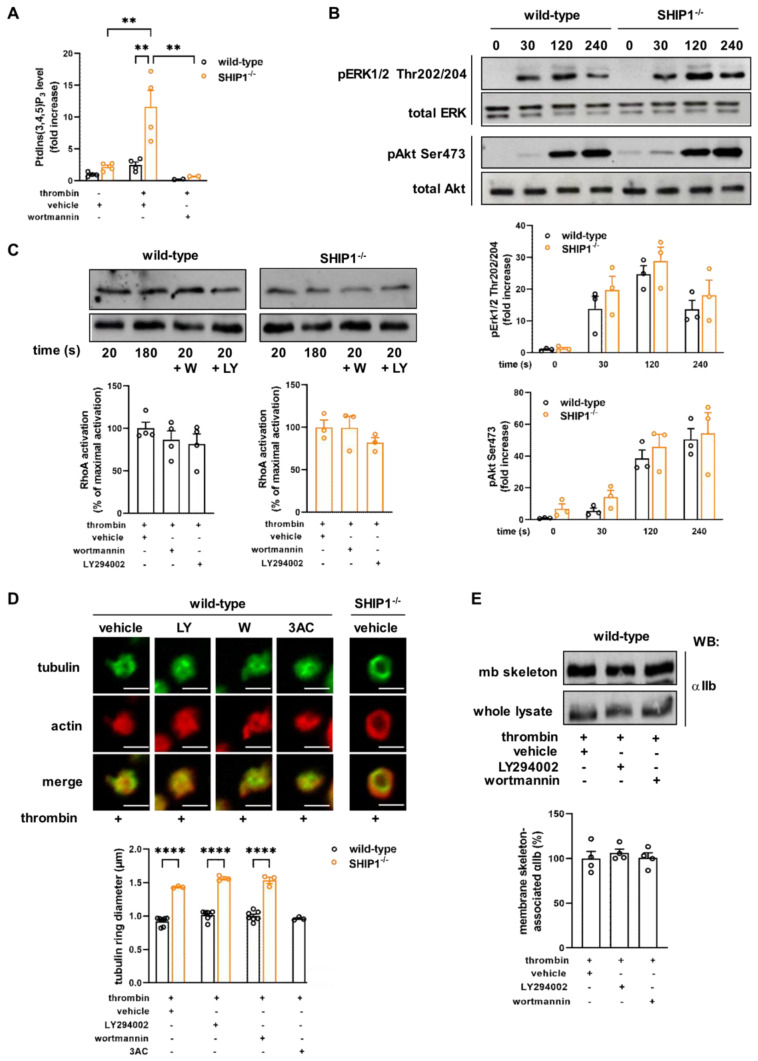
SHIP1 phosphatase activity is dispensable for Akt and ERK1/2 phosphorylation, RhoA activation, internal platelet contraction, and α_IIb_ integrin relocalization to the membrane skeleton. (**A**) Wild-type or SHIP1-deficient platelets were preincubated with vehicle (DMSO) or wortmannin (50 nM) and stimulated or not with thrombin (0.5 IU/mL, 3 min) at 37 °C in suspension under non-aggregating conditions. PtdIns(3,4,5)P_3_ production was quantified by mass spectrometry. Data are expressed as fold increase in vehicle-treated resting wild-type platelets, and are means ± SEM of 4 independent experiments. ** *p* < 0.01 according to two-way ANOVA test. (**B**) Wild-type and SHIP1-deficient platelets were stimulated with thrombin (0.5 IU/mL) in suspension under non-aggregating conditions at indicated times. Platelet lysates were then analyzed by Western blotting using phosphospecific antibodies against Erk1/2 (anti-pErk1/2 Thr202/204) and Akt (anti-pAkt Ser473). Anti–Erk and anti-Akt antibodies were used as loading controls. Representative Western-blots of 3 independent experiments are shown. Data are expressed as fold increase in resting (0 s) wild-type and are means ± SEM of 3 independent experiments. (**C**–**E**) Wild-type and SHIP1-deficient platelets were preincubated for 15 min in the presence of vehicle (DMSO), wortmannin (W, 50 nM), LY294002 (LY, 25 µM) or SHIP1 inhibitor 3AC (100 µM) when indicated, then stimulated by thrombin (0.5 IU/mL; (**D**) 2 min, (**E**) 4 min) in suspension under non-aggregating conditions. (**C**) The activated form of RhoA was analyzed. Representative Western blots of RhoA from RhoA-GTP pull-down and from whole lysates (total RhoA) are shown. Data are expressed as percentage of RhoA maximal activation of thrombin (20 s) condition and are means ± SEM of 3 independent experiments. (**D**) α-tubulin and actin reorganization were analyzed by confocal microscopy. Data of tubulin ring diameter are means ± SEM (*n* = 3–7 independent experiments). **** *p* < 0.0001 according to two-way ANOVA test. (**E**) After isolation of membrane skeleton from wild-type platelet, the amount of α_IIb_ integrin subunit was analyzed by Western blotting. Representative Western blots of α_IIb_ integrin subunit in the membrane (mb) skeleton fraction and in whole lysates are shown. Data are expressed as a percentage of vehicle and are means ± SEM of 4 independent experiments.

SHIP1 is the main phosphatase implicated in the regulation of PtdIns(3,4,5)P_3_ level in platelet aggregating conditions [15]. To investigate whether SHIP1 was also activated independently of aggregation, wild-type and SHIP1-deficient platelets were stimulated by thrombin in non-aggregating conditions, and the PtdIns(3,4,5)P_3_ level was quantified by mass spectrometry. Our results showed that SHIP1 was activated under these conditions since its absence induced an accumulation of PtdIns(3,4,5)P_3_ in thrombin-stimulated conditions, which was sensitive to the PI3K inhibitor, wortmannin (Figure 5A). Surprisingly, as shown in Figure 5B, the phosphorylation of the serine/threonine kinase Akt, a well-known effector of PtdIns(3,4,5)P_3_, and of the extracellular signal-regulated kinase 1/2 (Erk1/2) were not significantly increased by SHIP1 deficiency in platelets.

PtdIns(3,4,5)P_3_ accumulation in SHIP1-deficient platelets raised the question of whether the defect of cytoskeleton and integrin dynamics would result from the absence of SHIP1 phosphatase activity or from its docking proprieties. To answer this question, RhoA activation and the tubulin ring diameter were investigated in the presence of two unrelated PI3K inhibitors, wortmannin and LY294002 (Figure 5C,D). The results showed that PI3K inhibition did not reverse the effect of SHIP1 deficiency on thrombin-induced RhoA activation (Figure 5C), nor did it do so on the tubulin ring diameter (Figure 5D and Figure S4), suggesting that the reduction in RhoA activation in these platelets was not due to the accumulation of PtdIns(3,4,5)P_3_. In addition, inhibition of SHIP1-phosphatase activity by the 3AC compound [34] did not affect the tubulin ring diameter in wild-type platelets (Figure 5D). Together, these results support a PtdIns(3,4,5)P_3_ and phosphatase activity-independent role of SHIP1 in RhoA activation and cytoskeleton organization in platelets. In line with these results, the localization of α_IIb_ integrins to the membrane (mb) skeleton was also unaffected by wortmannin or LY294002 in wild-type platelets (Figure 5E).

Overall, these data point to a role for the docking functions of SHIP1 in the regulation of Rho-kinase/myosin IIA activation, a critical step for the cytoskeletal organization, integrin anchoring, and internal platelet contraction following thrombin stimulation during the first step of platelet activation.

## 3. Discussion

During the hemostatic response, platelets undergo a complex series of morphological changes that require dynamic remodeling of the cytoskeleton and generation of internal contractile forces. The function of the platelet contractile mechanism in regulating fibrin clot retraction is well documented [5,35] and several reports suggest an important role of this apparatus in the early phases of platelet responses during hemostasis [7,8,9,36]. Early platelet activation is associated with the internal platelet contraction [37] where actin–myosin IIA complexes are responsible for the contraction of the microtubule coil in platelets [28,36,38].

Here, we show that the absence of SHIP1 affects actomyosin complex formation and internal platelet contraction upon thrombin stimulation under non-aggregating conditions, independently of the PI3K/Akt pathway. This defect in the internal platelet contraction is linked to a decrease in the activation of the RhoA/Rho-kinase pathway and a significant reduction in myosin IIA association with the platelet membrane/cytoskeleton. As PI3K inhibition has no effect on RhoA activation and internal contraction in wild-type and SHIP1-deficient platelets, we propose that PtdIns(3,4,5)P_3_ is not impacting these processes and that SHIP1 mediates this effect via its scaffolding capacities.

In absence of SHIP1, RhoA activation is significantly decreased. The relocation of myosin IIA to the actin cytoskeleton is also affected in SHIP1-null platelets, and in wild-type platelets treated with the Rho-kinase inhibitor Y27632. This inhibitor has no additional effect when added to SHIP1-deficient platelets. This result supports the idea that in SHIP1-null platelets, decreased activation of the RhoA/Rho-kinase pathway is responsible for abnormal myosin IIA relocation to the membrane/cytoskeleton fraction and, in turn, for the deficiency of the internal platelet force. Rho-kinase proteins are Rho-GTPase-activated serine/threonine kinases that function as modulators of actomyosin cytoskeletal dynamics via the phosphorylation of several cytoskeleton-regulating proteins including myosin light chain (MLC) [39] but also Ezrin/Radixin/Moesin (ERM) proteins [40], myristoylated alanine-rich C-kinase substrate (MARCKS) [39] or LIM-kinase [41]. Deregulation of one of these proteins could explain the impact of Rho-kinase inhibition on myosin IIA localization and actomyosin complex formation. Consistent with this, we previously showed that phosphorylation of MLC was affected in SHIP1-deficient platelets at early phases of activation [27].

The platelet plasma membrane is lined by the spectrin-based membrane skeleton which impacts membrane properties such as shape, stability, and the lateral distribution of membrane glycoproteins. The membrane skeleton contains short actin filaments cross-linked by actin-binding proteins such as spectrin, vinculin, paxillin, or talin, providing an important platform for functional proteins to organize signaling complexes [1,42]. In unstimulated and stimulated platelets, the spectrin-based membrane skeleton is connected to the actin filaments present throughout the cytoplasm [43,44]. Our results indicate that SHIP1 is a component of the spectrin-based membrane skeleton and is required for the stable anchorage of α_IIb_ and β_1_ integrin subunits to this network before the aggregation process. Again, the fact that PI3K inhibition does not affect the localization of α_IIb_ integrin subunit strongly suggests that SHIP1 plays this role via its docking properties.

It has previously been described that the subpopulation of α_IIb_β_3_ integrins associated with the spectrin-based membrane skeleton is preferentially activated and subsequently incorporated into complexes with cytoplasmic actin filaments to form integrin-rich clusters [45,46]. Basically, inside-out signaling first modulates the affinity of the integrin to its ligand, which implies a structural change in the integrin, and then increases the avidity of the integrin for its ligand, which occurs through integrin clustering. These clusters play a role in stabilizing the ligand-integrin interactions, inducing a selective redistribution of occupied integrins and the subsequent outside-in signaling, facilitating the formation of mature focal adhesions, and increasing the aggregation strength. Previous studies highlighted the role of SHIP1 downstream of integrin activation and engagement [15,47,48]. Here, we show that during the early step of platelet activation, SHIP1 is an important regulator of α_IIb_β_3_ integrin organization into clusters via the control of stable integrin anchoring to the membrane skeleton and the contractile cytoskeleton. The absence of these mechanisms in SHIP1-null platelets may contribute to explain the defective platelet-platelet close contacts and aggregation upon stimulation despite normal fibrinogen binding and integrin activation [27].

Altogether, based on these new data and those from the literature, we propose that two distinct spatiotemporal roles for SHIP1 co-exist in platelets. Cytosolic SHIP1 is recruited to focal adhesions where it becomes tyrosine phosphorylated and transforms PtdIns(3,4,5)P_3_ into PtdIns(3,4)P_2_ following aggregation allowing RASA3/RAP1B regulation and α_IIb_β_3_ integrin downregulation [15,25]. Another pool of SHIP1 acts via its docking properties in the regulation of the RhoA/Rho-kinase pathway and the anchoring of both myosin IIA and α_IIb_β_3_ integrins to the cytoskeleton. This later function appears necessary for integrin clustering and transmission of internal platelet contractile forces during the early step of platelet activation.

## 4. Materials and Methods

Materials. Alexa fluor 488 goat anti-mouse or -rabbit antibodies and Alexa fluor 594 phalloidin were purchased from Invitrogen (Waltham, MA, USA). Y27632, blebbistatin, SHIP1 inhibitor 3AC were from Calbiochem (San Diego, CA, USA). Integrilin^®^ (eptifibatide) was purchased from GlaxoSmithKline (London, UK). Anti-pErk1/2 Thr202/204, anti-Erk1/2, anti-pAkt Ser473, anti-Akt, and anti-β3 antibodies were purchased from Cell Signaling Technology (Danvers, MA, USA). Anti-RhoA, anti-β1 and anti-α_IIb_ antibodies were purchased from Santa Cruz Biotechnology Inc (Dallas, TX, USA). Peroxidase-conjugated secondary antibodies were purchased from Promega (Madison, WI, USA). Fluorescein isothiocyanate (FITC)-labeled α_IIb_ and α_2_ antibodies were from BD Biosciences (Franklin Lakes, NJ, USA). All other antibodies and reagents were purchased from Sigma-Aldrich (Saint Louis, MI, USA) unless otherwise indicated.

Animals. Wild-type and SHIP1-deficient mice [20] were of C57BL/6/Sv129J genetic background. Mice were housed in the Anexplo (Toulouse) vivarium according to institutional guidelines. For all experiments, 8-to-14-week-oldmale or female mice were used. Ethical approval for animal experiments was obtained from the French Ministry of Research in agreement with European Union guidelines.

Preparation of murine platelets. Whole blood was drawn from the inferior vena cava of anesthetized mice into a syringe containing acid citrate dextrose (1 volume anticoagulant/9 volumes blood). Platelets were prepared as previously described [27].

Immunofluorescence microscopy. In this stage, 1 × 10^8^ washed platelets were preincubated with integrilin^®^ (40 µg/mL) for 5 min to prevent aggregation and when indicated with different inhibitors. Washed platelets were then stimulated or not with 0.5 IU/mL of thrombin, at 37 °C, in suspension and fixed after 2 min. After a centrifugation at 1000× *g* for 6 min, platelets were gently resuspended in PBS and allowed to settle on poly-L-lysine-coated coverslips for 30 min, at 37 °C. All incubations and washes were performed in 1% PBS-bovine serum albumin (BSA), at room temperature. Settled platelets were permeabilized with 0.1% Triton X-100 and incubated with the anti-α-tubulin antibody and, after washing, with Alexa fluor 488 goat anti-mouse antibody and Alexa fluor 594 phalloidin for actin labelling. Samples were mounted and examined using a LSM 510 or 710 laser scanning confocal microscope (Zeiss, Jena, Germany). To quantify the tubulin ring diameter, the linescan function of the Zeiss LSM software (Zeiss, Jena, Germany) was used as described [28,49]. Briefly, one reference line was drawn at the center of each platelet (as exemplified in Figure 1A(i–l)), and the software calculated the mean red and green fluorescence intensities along the reference lines then plotted the measurements. The distance (µm) between the two green peaks corresponds to the diameter of the tubulin ring. Overall, 10 to 40 platelets were analyzed per experiment.

Determination of activated cellular RhoA. A total of 2.5 × 10^8^ washed platelets were preincubated with integrilin^®^ (40 µg/mL) for 5 min to prevent aggregation and when indicated with different inhibitors. Platelets were then stimulated or not with 0.5 IU/mL of thrombin, at 37 °C, for indicated time in suspension and lysed at indicated times in a buffer containing 50 mM Tris pH 7.2, 500 mM NaCl, 10 mM MgCl_2_, 1% Triton X-100, 0.5% sodium deoxycholate, 0.1% SDS, 10 μg/mL leupeptin and 10 μg/mL aprotinin, and 1 mM phenylmethylsulfonyl fluoride. RhoA activation was determined by affinity purification (pull-down) with a fusion protein consisting of GST and the Rho-binding domain of Rhotekin (RBD) as previously described [50].

Cytosol depletion. A total of 5 × 10^7^ washed platelets were preincubated with integrilin^®^ (40 µg/mL) for 5 min to prevent aggregation and when indicated with different inhibitors. Platelets were then stimulated or not with 0.5 IU/mL of thrombin, at 37 °C, in suspension for indicated times, centrifuged (3000× *g* for 30 s) and resuspended in 20 mM PIPES buffer (pH 6.8) containing 150 mM KCl, 2 mM EDTA and 30 µg/mL saponin. After 5 min of shaking, at room temperature, the supernatant (cytosol) and the pellet (membranes and cytoskeleton) fractions were separated by centrifugation (12,000× *g* for 40 s) and resuspended in electrophoresis sample buffer containing 100 mM Tris-HCl (pH 6.8), 15% glycerol, 25 mM DTT and 3% SDS for SDS-PAGE.

Isolation of cytoskeleton and membrane skeleton. In this stage, 1 × 10^8^ washed platelets were preincubated with integrilin^®^ (40 µg/mL) for 5 min to prevent aggregation and when indicated with different inhibitors. Platelets were then stimulated or not with 0.5 IU/mL of thrombin, at 37 °C, in suspension, and the cytoskeleton was extracted as previously described [51]. Briefly, platelets were lysed by the addition of ice-cold cytoskeleton buffer containing 100 mM Tris-HCl, pH 7.4, 20 mM EGTA, 2% Triton X-100, 2 mM Na_3_VO_4_, 4 μg/mL aprotinin, 4 μg/mL leupeptin, 2 mM phenylmethylsulfonyl fluoride. Cytoplasmic actin filaments were immediately sedimented by centrifugation at 15,600× *g* for 4 min and lysed in electrophoresis sample buffer for SDS-PAGE and coomassie blue staining. The platelet membrane skeleton was isolated from the 15,600× *g* supernatant by centrifugation at 100,000× *g* overnight in 1.5 mL conical microfuge tubes in a Beckman TL100 centrifuge using a TLA100.4 rotor (Beckman Coulter Life Sciences, Indianapolis, IN, USA) [52]. The Triton X-100-insoluble fraction obtained was solubilized in 2× ice electrophoresis sample buffer for SDS-PAGE.

Flow cytometry. A total of 1 × 10^6^ platelets were stained with FITC-conjugated anti–mouse a2 and anti–mouse GPIIb for 30 min and analyzed using an LSRFortessaTM Cell analyser flow cytometer and Diva software (Becton Dickinson, Franklin Lakes, NJ, USA).

Platelet surface protein cross-linking. Here, 3 × 10^7^ platelets were preincubated with integrilin^®^ (40 µg/mL) for 5 min and stimulated or not with 0.5 IU/mL of thrombin, at 37 °C, in suspension. Then, 0.25 mM Sulfo-EGS was added or not to platelet suspensions for 15 min, at room temperature. The reaction was then quenched with the addition of 25 mM Tris-HCl pH 7.5 for a further 10 min, at room temperature. Platelets were then lysed with the addition of an equal volume of 2x ice electrophoresis sample buffer for SDS-PAGE.

Gel electrophoresis and immunoblotting. Protein extracts were loaded on polyacrylamide gels, separated by SDS-PAGE and transferred on a nitrocellulose membrane (Gelman Sciences). The membrane was blocked using 3% BSA in Tris-Buffer-Saline containing 0.1% Tween (TBST) for 60 min. Membranes were probed overnight with the relevant primary antibody, washed with TBST, and probed with respective peroxidase-conjugated secondary antibodies for 1 h. Signals were then detected using ECL system. The various bands were quantified by densitometric analysis using Image J software.

Phospholipid extraction and analysis. In this stage, 1 × 10^8^ platelets (200 µL) were preincubated with integrilin^®^ (40 µg/mL) for 5 min and stimulated or not with 0.5 IU/mL of thrombin, at 37 °C, in suspension. The stimulation was stopped by the addition of 750 μL of a quench mixture composed of 484 mL of CH_3_OH, 242 mL of CHCl_3_, and 23.55 mL of 1 M HCl, and lipids were immediately extracted and derivatizated by TMS-diazomethane as previously described. Mass spectral analysis was performed on LC-QqQ triple quadrupole mass spectrometer (LC-vcQQQ 6460 Agilent) equipped with electrospray ionization operating positive mode. Analyses were performed in Selected Reaction Monitoring detection mode (SRM) using nitrogen as collision gas [53]. Finally, peak detection, integration and quantitative analysis were performed using MassHunter QqQ Quantitative analysis software (Agilent Technologies VersionB.05.00). Data were represented as ratio area sample/area internal standard per 1 × 10^6^ platelets and expressed as fold increase.

Statistical analysis. Data are expressed as means ± S.E.M. Statistical analysis were performed using GraphPad Prism version 9.3.1 (San Diego, CA, USA). Normality and homogeneity of variance were assessed by Shapiro–Wilk and Brown–Forsythe tests, respectively. After confirming homogeneous variances and normality, group comparisons for means were performed by unpaired student t test or one- or two-way analysis of variance (ANOVA) test with Holm–Sidak multiple comparison test. *p* < 0.05 was considered statistically significant.

## Data Availability

Not applicable.

## Supplementary Materials

The following supporting information can be downloaded at: https://www.mdpi.com/article/10.3390/ijms24020958/s1.
