# Supplementary material for: SHIP1 Controls Internal Platelet Contraction and αIIbβ3 Integrin Dynamics in Early Platelet Activation
Source: Int J Mol Sci. 2023 Jan 4;24(2):958. doi: 10.3390/ijms24020958 (PMC9860818; doi:10.3390/ijms24020958)
Supplement: Supplementary file 1 [file ijms-24-00958-s001.zip › Supplemental Figures Severin et al.pdf]

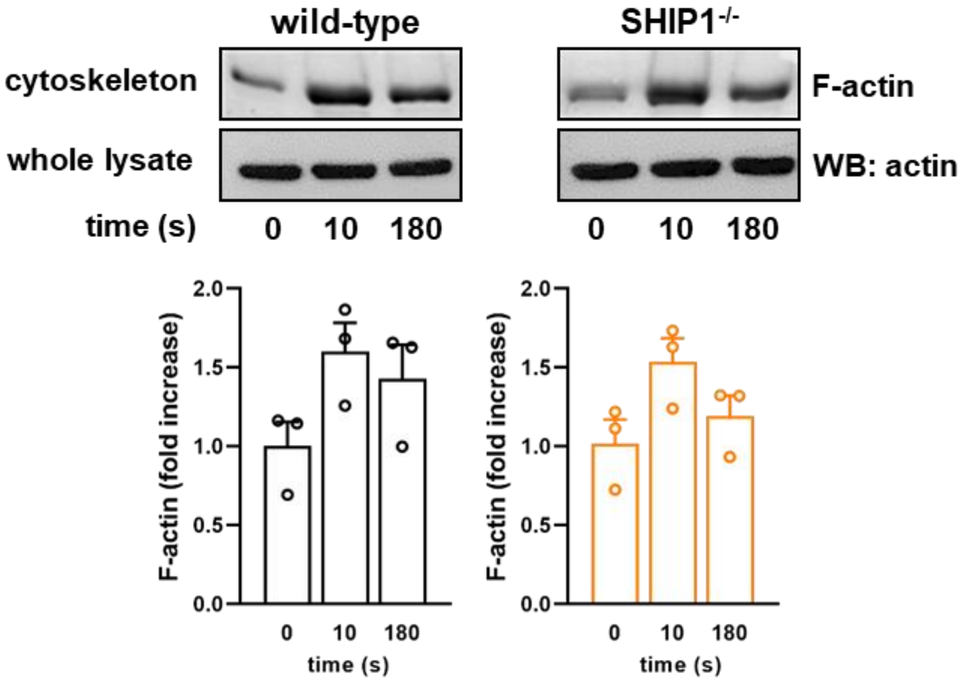

Figure S1 : SHIP1 is dispensable for actin polymerisation in thrombin-stimulated platelets. Wild-type and SHIP1-deficient platelets were activated with thrombin (0.5 IU/ml) in suspension under non-aggregating conditions before isolation of their cytoskeleton to quantify the amount of F-actin. Representative coomassie staining of F-actin and Western blots of actin on whole lysates were shown. Data are expressed as fold increase of resting (0 s) and are means ± SEM of 3 independent experiments.

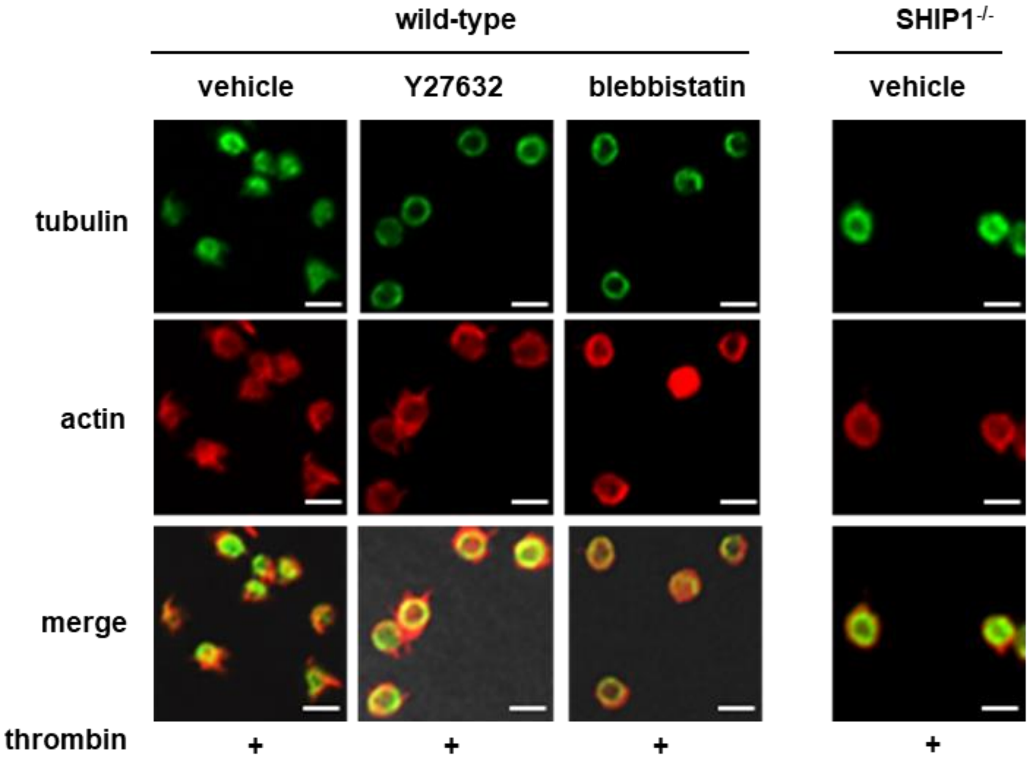

Figure S2 : Inhibition of Rho-kinase or myosin IIA activity affects thrombin-induced internal contraction in platelets. Wild-type and SHIP1-deficient platelets were preincubated with vehicle (DMSO), 20 µM Y27632 or 10 µM blebbistatin for 20 min before thrombin stimulation (0.5 IU/ml) for 2 min under non-aggregating suspension conditions. Representative confocal images of platelet microtubule (green) and actin cytoskeleton (red) from 4 independent experiments are shown (scale bar = 2 µm).

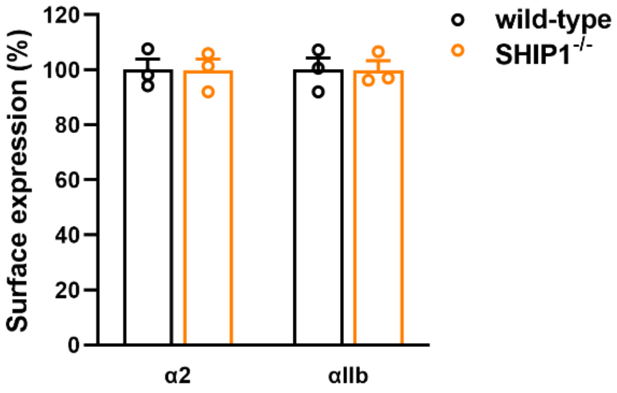

Figure S3 : Similar expression of integrin subunits on the surface of wild-type and SHIP1-deficient platelets. Platelet surface expression of α2 and αIIb integrin subunits was determined by flow cytometry using specific fluorescein isothiocyanate (FITC)-labeled antibodies. Results are expressed as relative percentage of surface expression and are means ± SEM of 3 independent experiments,

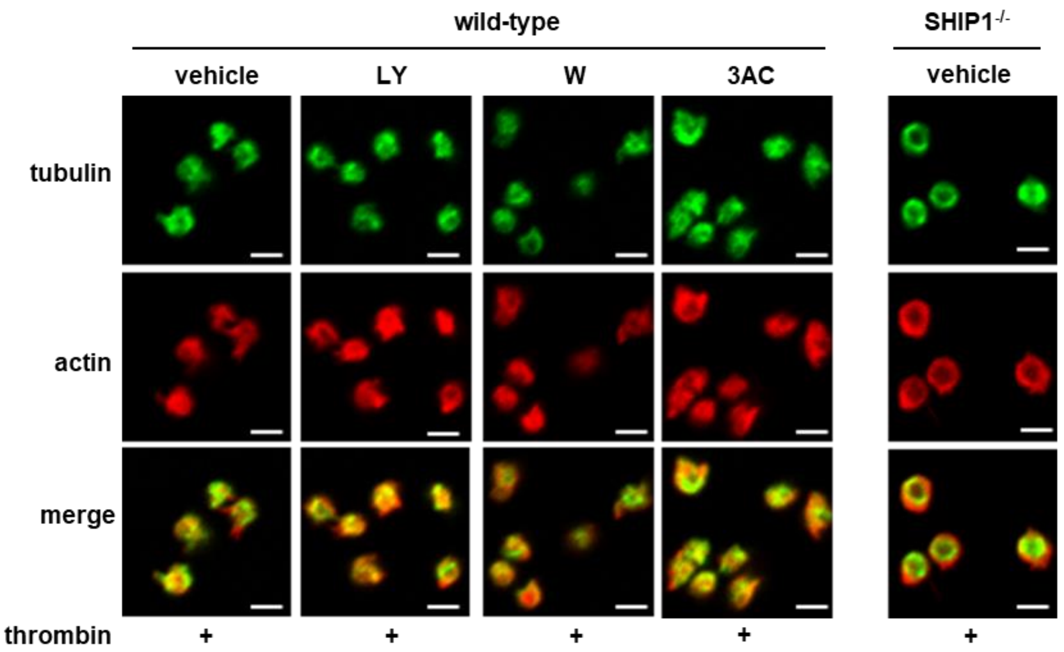

Figure S4 : SHIP1 phosphatase activity is dispensable for internal platelet contraction. Wild-type and SHIP1-deficient platelets were preincubated for 15 min in the presence of vehicle (DMSO), LY294002 (LY, 25 µM), wortmannin (W, 50 nM) or SHIP1 inhibitor 3AC (100 µM), then stimulated by thrombin (0.5 IU/ml) in suspension under non-aggregating conditions. Representative confocal images of platelet microtubule (green) and actin cytoskeleton (red) from 4 independent experiments are shown (scale bar = 2 µm).
